# Examining barriers to implementing a surgical-site infection bundle

**DOI:** 10.1017/ice.2023.114

**Published:** 2024-01

**Authors:** Kimberly C. Dukes, Heather Schacht Reisinger, Marin Schweizer, Melissa A. Ward, Laura Chapin, Timothy C. Ryken, Trish M. Perl, Loreen A. Herwaldt

**Affiliations:** 1 Center for Access & Delivery Research & Evaluations (CADRE), Iowa City Veterans’ Affairs (VA) Health Care System (ICVAHCS), Iowa City, Iowa; 2 Carver College of Medicine, University of Iowa, Iowa City, Iowa; 3 College of Public Health, University of Iowa, Iowa City, Iowa; 4 Institute for Clinical and Translational Science, University of Iowa, Iowa City, Iowa; 5 Department of Internal Medicine, School of Medicine and Public Health, University of Wisconsin, Madison, Wisconsin; 6 Boston Scientific, Marlborough, Massachusetts; 7 MercyOne Northeast Iowa Neurosurgery, Iowa City, Iowa; 8 Department of Neurosurgery, Dartmouth-Hitchcock Medical Center, Lebanon, New Hampshire; 9 University of Texas Southwestern Medical Center, Dallas, Texas; 10 University of Iowa Hospitals and Clinics (UIHC), Iowa City, Iowa

## Abstract

**Background:**

Surgical-site infections (SSIs) can be catastrophic. Bundles of evidence-based practices can reduce SSIs but can be difficult to implement and sustain.

**Objective:**

We sought to understand the implementation of SSI prevention bundles in 6 US hospitals.

**Design:**

Qualitative study.

**Methods:**

We conducted in-depth semistructured interviews with personnel involved in bundle implementation and conducted a thematic analysis of the transcripts.

**Setting:**

The study was conducted in 6 US hospitals: 2 academic tertiary-care hospitals, 3 academic-affiliated community hospitals, 1 unaffiliated community hospital.

**Participants:**

In total, 30 hospital personnel participated. Participants included surgeons, laboratory directors, clinical personnel, and infection preventionists.

**Results:**

Bundle complexity impeded implementation. Other barriers varied across services, even within the same hospital. Multiple strategies were needed, and successful strategies in one service did not always apply in other areas. However, early and sustained interprofessional collaboration facilitated implementation.

**Conclusions:**

The evidence-based SSI bundle is complicated and can be difficult to implement. One implementation process probably will not work for all settings. Multiple strategies were needed to overcome contextual and implementation barriers that varied by setting and implementation climate. Appropriate adaptations for specific settings and populations may improve bundle adoption, fidelity, acceptability, and sustainability.

Surgical site infections (SSIs) can be catastrophic for patients^1,2^ and expensive for hospitals.^3–6^ The US Department of Health and Human Services set a goal of reducing SSIs by 30% by 2020.^7^ To achieve this goal, healthcare institutions have developed and implemented bundles of evidence-based practices.^8–11^ However, evidence-based bundles can be difficult to implement and sustain due to complex bundles, patient and process variation, poor compliance, workflow and communication obstacles, and other barriers.^12–14^ Qualitative and implementation science approaches provide important perspectives regarding implementation of infection prevention measures, including care bundles.

The Study to Optimally Prevent SSIs in Select Cardiac and Orthopedic Procedures (STOP SSI) tested a bundle that included (1) screening patients for methicillin-susceptible and methicillin-resistant *Staphylococcus aureus* (MSSA and MRSA), (2) decolonizing *S. aureus* carriers, (3) giving MRSA carriers and patients whose carrier status was unknown vancomycin and cefazolin as perioperative prophylaxis, and (4) providing chlorhexidine baths for noncarriers. Full adherence to the bundle significantly reduced the incidence of *S. aureus* among patients undergoing cardiac operations or total hip or total knee arthroplasty at 20 Hospital Corporation of America (HCA)–affiliated hospitals.^15^

To successfully implement infection prevention bundles like STOP SSI, hospitals must identify factors that enable or obstruct successful bundle implementation. To examine barriers and facilitators of SSI-prevention bundles at diverse hospitals, we interviewed healthcare personnel who played key roles in bundle implementation at 2 hospitals that implemented the full STOP SSI bundle as well as 4 hospitals that implemented similar steps without screening. We analyzed the data using the Consolidated Framework for Implementation Research (CFIR) framework.

## Methods

We conducted a qualitative evaluation to examine the contextual factors that influenced bundle adoption and implementation. We purposefully sampled healthcare personnel (HCP) who helped implement the bundle and conducted in-depth, semistructured interviews with 30 HCP (22 at academic hospitals, 8 at community hospitals). We first defined key informant roles as surgeons, laboratory personnel, hospital epidemiologists, infection preventionists, and clinic staff involved in implementation. Relevant staff roles differed across sites, and interview data demonstrated that other processes were important. Therefore, we interviewed a day-of-surgery-admission (DOSA) nurse, a pharmacist, and 2 anesthesiologists to identify further perspectives.

### Settings

We interviewed staff at 2 academic, tertiary-care hospitals; 3 academic-affiliated, community hospitals; and 1 unaffiliated community hospital in the US Mid-Atlantic and Midwest regions. Two hospitals implemented the full STOP SSI bundle and 4 hospitals implemented similar steps without screening patients. None of these hospitals participated in the original STOP SSI study. Surgical services varied by hospital but included cardiothoracic surgery, orthopedic surgery, and neurosurgery. To safeguard confidentiality for participants and institutions, we report only the participants’ roles and the hospital type.

### Data collection

A medical anthropologist (K.D.) conducted interviews in person or by phone; 29 interviews were recorded and transcribed. The interviewer took detailed notes during one interview because the participant declined to be recorded. We imported transcripts into MAXQDA10,^16^ a qualitative data analysis management software.

### Data analysis

To conduct thematic analysis,^17^ the team employed 2 phases. First, 2 medical anthropologists (K.D. and H.S.R.) read a subset of 4 transcripts and developed a consensus codebook including a priori codes defined by research questions and inductive codes that emerged during analysis. They coded 2 transcripts together to ensure agreement. K.D. then coded the remaining transcripts. Subsequently the team mapped codes in the initial codebook to constructs, or factors, using the CFIR developed by Damschroder et al^18^ to provide a comprehensive set of constructs distilled from a range of implementation models. Principal CFIR constructs include inner and outer settings, processes, characteristics of the intervention, and characteristics of individuals involved in the intervention. Detailed definitions of all CFIR constructs and subconstructs are available.^18^ As the team mapped their existing codes onto CFIR constructs, they identified 3 CFIR constructs most relevant to interpreting our data: intervention characteristics, inner setting, and process. Within each of these constructs, we also identified specific subconstructs that were most relevant. We have reported the connection of our results with CFIR constructs and subconstructs in Table 1. Additionally, using CFIR tools,^19,20^ we identified implementation barriers (Table 2) and evidence-based strategies that may assist implementation (Table 3).^21^

**Table 1.** An Adaptation of the Consolidated Framework for Implementation Research (CFIR) Constructs and Subconstructs^18^

Construct and Subconstructs		Short Description	Theme Number
**I.**	**Intervention characteristics**		**1**
A	Intervention source	Perception of key stakeholders about whether the intervention is externally or internally developed.	
B	Evidence strength and quality	Stakeholders’ perceptions of the quality and validity of evidence supporting the belief that the intervention will have desired outcomes.	
C	Relative advantage	Stakeholders’ perception of the advantage of implementing the intervention versus an alternative solution.	
D	**Adaptability**	**The degree to which an intervention can be adapted, tailored, refined, or reinvented to meet local needs.**	**1**
E	**Trialability**	**The ability to test the intervention on a small scale in the organization, and to be able to reverse course (undo implementation) if warranted.**	**1**
**F**	**Complexity**	**Perceived difficulty of the intervention, reflected by duration, scope, radicalness, disruptiveness, centrality, and intricacy and number of steps required to implement.**	**1**
G	**Design quality and packaging**	**Perceived excellence in how the intervention is bundled, presented, and assembled.**	**1**
H	Cost	Costs of the intervention and costs associated with implementing the intervention including investment, supply, and opportunity costs.	
**II.**	**Outer setting**		
A	Patient needs and resources	The extent to which patient needs, as well as barriers and facilitators to meet those needs, are accurately known and prioritized by the organization.	
B	Cosmopolitanism	The degree to which an organization is networked with other external organizations.	
C	Peer pressure	Mimetic or competitive pressure to implement an intervention; typically because most or other key peer or competing organizations have already implemented or are in a bid for a competitive edge.	
D	External policies and incentives	A broad construct that includes external strategies to spread interventions, including policy and regulations (governmental or other central entity), external mandates, recommendations and guidelines, pay-for-performance, collaboratives, and public or benchmark reporting.	
**III.**	**Inner setting**		
**A**	**Structural characteristics**	**The social architecture, age, maturity, and size of an organization.**	**2**
**B**	**Networks and communications**	**The nature and quality of webs of social networks and the nature and quality of formal and informal communications within an organization.**	**2**
**C**	**Culture**	**Norms, values, and basic assumptions of a given organization.**	**2**
**D**	**Implementation climate**	**The absorptive capacity for change, shared receptivity of involved individuals to an intervention, and the extent to which use of that intervention will be rewarded, supported, and expected within their organization.**	**2**
1	**Tension for change**	**The degree to which stakeholders perceive the current situation as intolerable or needing change.**	**2**
2	**Compatibility**	**The degree of tangible fit between meaning and values attached to the intervention by involved individuals, how those align with individuals’ own norms, values, and perceived risks and needs, and how the intervention fits with existing workflows and systems.**	**2**
3	**Relative priority**	**Individuals’ shared perception of the importance of the implementation within the organization.**	**2**
4	Organizational incentives and rewards	Extrinsic incentives such as goal-sharing awards, performance reviews, promotions, and raises in salary, and less tangible incentives such as increased stature or respect.	
5	Goals and feedback	The degree to which goals are clearly communicated, acted upon, and fed back to staff, and alignment of that feedback with goals.	
6	Learning climate	A climate in which (a) leaders express their own fallibility and need for team members’ assistance and input; (b) team members feel that they are essential, valued, and knowledgeable partners in the change process; (c) individuals feel psychologically safe to try new methods; and (d) there is sufficient time and space for reflective thinking and evaluation.	
**E**	**Readiness for implementation**	**Tangible and immediate indicators of organizational commitment to its decision to implement an intervention.**	**2**
1	**Leadership engagement**	**Commitment, involvement, and accountability of leaders and managers with the implementation.**	**2**
2	**Available resources**	**The level of resources dedicated for implementation and ongoing operations, including money, training, education, physical space, and time.**	**2**
	**Access to knowledge and information**	**Ease of access to digestible information and knowledge about the intervention and how to incorporate it into work tasks.**	**2**
**IV.**	**Characteristics of individuals**		
A	Knowledge and beliefs about the intervention	Individuals’ attitudes toward and value placed on the intervention as well as familiarity with facts, truths, and principles related to the intervention.	
B	Self-efficacy	Individual belief in their own capabilities to execute courses of action to achieve implementation goals.	
C	Individual stage of change	Characterization of the phase an individual is in, as he or she progresses toward skilled, enthusiastic, and sustained use of the intervention.	
D	Individual identification with organization	A broad construct related to how individuals perceive the organization, and their relationship and degree of commitment with that organization.	
E	Other personal attributes	A broad construct to include other personal traits such as tolerance of ambiguity, intellectual ability, motivation, values, competence, capacity, and learning style.	
**V.**	**Process**		**3**
**A**	**Planning**	**The degree to which a scheme or method of behavior and tasks for implementing an intervention are developed in advance, and the quality of those schemes or methods.**	**3**
**B**	**Engaging**	**Attracting and involving appropriate individuals in the implementation and use of the intervention through a combined strategy of social marketing, education, role modeling, training, and other similar activities.**	**3**
1	**Opinion leaders**	**Individuals in an organization who have formal or informal influence on the attitudes and beliefs of their colleagues with respect to implementing the intervention.**	**3**
2	**Formally appointed internal implementation leaders**	**Individuals from within the organization who have been formally appointed with responsibility for implementing an intervention as coordinator, project manager, team leader, or other similar role.**	**3**
3	**Champions**	**“Individuals who dedicate themselves to supporting, marketing, and ‘driving through’ an [implementation],”** ^**101(p.182)**^ **overcoming indifference or resistance that the intervention may provoke in an organization.**	**3**
4	External change agents	Individuals who are affiliated with an outside entity who formally influence or facilitate intervention decisions in a desirable direction.	
**C**	**Executing**	**Carrying out or accomplishing the implementation according to plan.**	**3**
D	**Reflecting and evaluating**	**Quantitative and qualitative feedback about the progress and quality of implementation accompanied with regular personal and team debriefing about progress and experience.**	**3**

Note. The constructs and descriptions most relevant to the analysis for this study appear in bold font, along with the theme number in which constructs are discussed. Construct titles and descriptions are by Damschroder et al.^18^

**Table 2.** Reported Relevant Barriers as Framed by the Consolidated Framework for Implementation Research-Expert Recommendations for Implementing Change (CFIR-ERIC) Barrier Buster Tool version 0.53^19^

Intervention Characteristics	CFIR Construct Definition Framed as a Barrier
Complexity	Stakeholders believe that the innovation is complex based on their perception of duration, scope, radicalness, disruptiveness, centrality, and/or intricacy and number of steps needed to implement.
Design quality and packaging	Stakeholders believe the innovation is poor quality based on the way it is bundled, presented, and/or assembled.
**Inner setting**	
Structural characteristics	The social architecture, age, maturity, and size of an organization hinders implementation.
Networks and communications	The organization has poor quality or non-productive social networks and/or ineffective formal and informal communications.
Culture	Cultural norms, values, and basic assumptions of the organization hinder implementation.
Implementation climate	There is little capacity for change, low receptivity, and no expectation that use of the innovation will be rewarded, supported, or expected.
Tension for change	Stakeholders do not see the current situation as intolerable or do not believe they need to implement the innovation.
Compatibility	The innovation does not fit well with existing workflows nor with the meaning and values attached to the innovation, nor does it align well with stakeholders’ own needs and/or it heightens risk for stakeholders.
Relative priority	Stakeholders perceive that implementation of the innovation takes a backseat to other initiatives or activities.
Readiness for implementation	There are few tangible and immediate indicators of organizational readiness and commitment to implement the innovation.
**Process**	
Planning	A scheme or sequence of tasks necessary to implement the intervention has not been developed or the quality is poor.
Formally appointed internal implementation leaders	A skilled implementation leader (coordinator, project manager or team leader), with responsibility to lead implementation of the innovation, has not been formally appointed or recognized within the organization.
Champions	Individuals acting as champions who support, market, or ‘drive through’ implementation in a way that helps to overcome indifference or resistance by key stakeholders are not involved or supportive.
Executing	Implementation activities are not being done according to plan.
Reflecting and evaluating	There is little or no quantitative and qualitative feedback about the progress and quality of implementation nor regular personal and team debriefing about progress and experience.

**Table 3.** Twelve Expert Recommendations for Implementing Change (ERIC) Strategies^21^

Recommended ERIC Strategies
Assess for readiness and identify barriers and facilitators
Develop a formal implementation blueprint
Identify and prepare champions
Conduct local consensus discussions
Promote adaptability
Facilitation
Capture and share local knowledge
Organize clinician implementation team meetings
Conduct local needs assessment
Develop and implement tools for quality monitoring
Tailor strategies

Note. Identified by the Consolidated Framework for Implementation Research- Expert Recommendations for Implementing Change (CFIR-ERIC) Barrier Buster Tool version 0.53^19^ that could help overcome or reduce barriers identified in Table 2.

The University of Iowa Institutional Review Board approved the study.

## Results

In total, 30 HCP participated in interviews. We identified 3 principal themes about implementation barriers and facilitators; they are discussed in the following sections.

### Theme 1. Complexity

The complexity of the intervention impeded implementation. Hardwiring through protocols and order sets as well as clear communication about the bundle itself reduced some barriers (Table 4).

The intervention’s complexity complicated both implementation and sustainment within surgical clinics and across the hospital. Participants noted that bundle fidelity required coordination with multiple hospital areas, including the laboratory and pharmacy, and across inpatient and outpatient domains with different processes for orders and documentation.

**Table 4.** Theme 1: The Complexity of the Intervention Itself Impeded Implementation in which Hardwiring Through Protocols and Order Sets, and Clear Communication About the Bundle Itself, Reduced Some Barriers.

Illustrative Quotes
“Everybody was kind of doing things different, so we formulated a protocol…”—Nurse, academic hospital “Our antibiotic recommendations are different for different surgeries. So it’s not standard enough that, they have a set order set.”—ID/hospital epidemiologist, academic hospital “This flow sheet for me has been a godsend. … It gives exact, ‘You do this, this, this, and this’ kind of instruction, whereas before you had to think ‘Oh gosh now what do I do if it’s this vs. this.’ This I keep with me in my surgery book at all times, it’s kind of like my little bible sheet.”—Nurse, academic hospital

Note. Quotes lightly edited for clarity.

Some HCP noted that each bundle step required them to remember to complete multiple processes (eg, check the status of swabs, results, notifications, prescriptions, and prophylaxis). Additionally, HCP reported that approaches to managing the complexity were varied and sometimes individualized, even within a single hospital. This variation made it difficult to ensure intervention fidelity. In addition to the complexity of the bundle itself, some groups within each hospital varied in their ability to implement bundle elements depending on varied rules and flexibility. For example, within one academic hospital, the staff members who were permitted to swab patients’ nares, contact patients about their carrier status, and order mupirocin or perioperative antibiotics varied by surgical service.

Many HCP reported that protocols or electronic order sets helped ensure fidelity across clinics and improved clinical workflow. At some hospitals, the availability and acceptability of order sets and protocols, residents’ or advanced practice providers’ work responsibilities, or access to informatics staff who could revise order sets varied by service. However, some departments could not establish or adapt protocols or order sets. One community hospital used paper-based presurgical orders. In addition, they had to use flexible approaches to overcome barriers. Approaches included advocating for new protocols, adapting other services’ existing protocols, or making another service’s order set a “favorite” in the electronic medical record.

However, bundle processes could be pilot tested and adapted, which facilitated the implementation process. Hospitals tailored processes within the institution and sometimes adapted processes for specific clinics. Several hospitals pilot tested the bundle in one surgical area before extending it to other areas or populations. Adding pieces of the protocol onto existing screening or decolonization processes also facilitated implementation.

The way the intervention was described and presented also affected implementation. One nurse coordinator used a flowsheet to review each patient and to ensure that she had not missed steps, but the fuzzy photocopy used at another hospital confused HCP.

### Theme 2. Implementation barriers

Implementation barriers varied with implementation climate and type of setting, and multiple strategies were required to overcome barriers (Table 5).

The hospital type affected the obstacles encountered. For example, academic hospital staff reported that surgical or anesthesiology residents or fellows often were unfamiliar with the bundle, and staff at a community hospital needed to persuade independent surgeons to accept the intervention. What CFIR describes as the implementation climate—including capacity for change, staff members’ receptivity, and organizational support for the intervention—also shaped implementation. For example, within a single hospital, HCP revealed that key personnel in one surgical area did not perceive a need to change processes, and thus resisted the intervention, while another surgical area had already integrated the bundle into their practice. Infection rates often influenced decisions of where to start the implementation or affected staff member’s willingness to change pre-existing practices. One academic hospital initially implemented the bundle on a specific surgical unit because the area had identified a number of SSIs. In contrast, surgeons in another specialty did not perceive a need to change practice. A few HCP noted that sometimes mandates were required. At one clinic that mandated bundle use, staff collaborated to overcome one surgeon’s resistance, partly by ensuring that he did not have to change his practice. A nurse practitioner commented, “We make it happen and he doesn’t have to really get involved at all.” At one community hospital, some patients were missed because a physician assistant and scheduler were reluctant to collaborate. Explicit surgical leadership support finally persuaded all staff to support full bundle implementation.

**Table 5.** Theme 2: Implementation Barriers Can Vary With Implementation Climate and Type of Setting, and Required Multiple Strategies to Try to Overcome Them

Illustrative Quotes
“We don’t have that kind of flexibility here, there’s a lot of resistance …. And we don’t have the authority to say “only our patients need to be asked these sets of questions.” … We’re not a teaching institution. … the frame of mind is a little bit different here.”—Infection preventionist, community hospital “We just walk in, make eye contact with someone, let them know that we’re dropping off the sample…”—Infection preventionist, community hospital “Once we have those two groups constantly sending samples down, it increases our workflow. … there are times where we have to let a sample sit because we’re already full.”—Microbiology laboratory technician, academic hospital …We are so big and every specialty is so different so the workflows are different so you kind of have to figure out how to do that. —Nurse, academic hospital “It’s just a basic difference between, uh you know uh different department uh, infrastructure. How much ancillary staff they have, and … how many mid-level providers are designated, for how many surgeons.” —ID physician, academic hospital “[There was] resistance … about doing the nasal swabs, and trying to kind of get the timing down [for MRSA carriers]… then trying to get the PAs [physician assistants] on board, [and] certifying two of our nurses to give nasal swabs. I felt a little frustrated.”—Infection preventionist, community hospital

Note. Quotes lightly edited for clarity.

Established communication pathways often helped staff implement this complex intervention but also differed across hospitals and services. For example, laboratory personnel noted that they could communicate relatively easily with clinics that had specific staff who guided patients’ preoperative evaluations or that identified specific staff members who received test results. In contrast, at one academic hospital, the laboratory could not deliver results to some clinics because staff members were not willing to receive them. Interviewees noted that coordinating across inpatient and outpatient settings often complicated implementation. Coordinating with diverse outpatient offices about swabbing and test results was sometimes difficult. However, dedicated staff facilitated coordination. At one community hospital, a dedicated nurse coordinator communicated with multiple affiliated surgeons from external offices. At an academic hospital, dedicated physician assistants facilitated implementation for outpatients on 2 surgical services. Staff at one academic hospital reported that implementation was more difficult on inpatient units due to the number and types of staff, different workflows and order protocols, and different practice standards.

Demonstrating a hospital’s readiness for implementation, HCP described engaging leaders, providing resources (money, training, education, space, and time), and ensuring access to knowledge and information. Some resources were provided once (eg, necessary laboratory equipment) but others were recurring needs. For example, staff turnover required hospitals to identify new champions and educate new personnel.

### Theme 3. Collaborative planning

Collaboration in planning, engaging, and executing implementation needed to begin before the intervention and to be sustained (Table 6).

Strong communication and collaboration improved the implementation process. Across hospitals, HCP emphasized the importance of thinking through the whole process and identifying every worker or hospital area who would be affected, including staff in laboratories, anesthesia, pharmacy, hospital stores and supply chains, and information technology, as well as HCP directly involved in surgical patient care.

**Table 6.** Theme 3: Collaboration in Planning, Engaging, and Executing Implementation Needed to Begin Before the Intervention and Need to be Sustained

Illustrative Quotes
“Make sure when you look at a surgical patient, who is everybody that’s gonna touch ‘em?”—Nurse, academic hospital “That’s pretty much with any sort of process change. You wanna be sure that everybody is aware of what you wanna do and why.”—Infection preventionist, community hospital “I think an important thing is to include all the areas that are gonna be involved.… If you’re gonna implement something that has to do with the lab, you need to be sure that they’re on board … [also] we have some strong surgeons and infectious disease physicians who people respect … That adds extra support and credibility.”—Infection preventionist, community hospital “Getting things in place, getting the people ready, …[getting] a test code for the lab… Making sure that someone’s actually gonna check the data and see what’s happening, like all those things always take a long time.”—ID/hospital epidemiologist, academic hospital “Like have you communicated with the lab, are they ok with processing these, or have you communicated with the department who normally does nasal swabs.”—Infection preventionist, community hospital

Note. Quotes lightly edited for clarity.

Participants who described relatively easy processes of implementation also reported early and successful collaboration in planning, engaging appropriate stakeholders and champions, and executing the implementation as planned. Early partners included information technology staff members (eg, to develop templates for preoperative visits or revise order sets), staff members across inpatient and outpatient settings (eg, to identify opportunities for swabbing patients), laboratory staff, and schedulers in other clinics (eg, to identify appropriate timeframes for sending swabs to the laboratory). Across hospitals, both formally appointed implementation leaders and formal or informal champions helped drive the implementation and overcome indifference and resistance. HCP across surgery types and hospital settings emphasized the importance of recruiting champions, persuading surgeons and other staff, and including all relevant stakeholders in planning to improve engagement and commitment. Commonly identified bundle champions included surgeons, nurses and nurse managers, hospital epidemiologists, and infection prevention staff or infectious disease specialists.

At times, staff had to begin the planning process well before the intervention. Interviewees described the importance of planning to both implement and sustain the protocol. Several stated that they needed to work with the infection prevention program or committee to implement the intervention. Laboratory leaders needed time to develop or adapt processes and have them approved, time to rearrange laboratory space to facilitate rapid sample testing, and in one case, time to write a grant proposal to buy appropriate equipment. HCP in both academic and community hospitals noted that they had to provide surgeons with evidence before they accepted the intervention.

HCP sometimes encountered obstacles because key stakeholders had not been identified (eg, pharmacy, anesthesiology at one hospital) or had not been included in planning (eg, laboratory personnel at another hospital). Planning sessions allowed important stakeholders to be integrated into the process early on. However, some institutions identified stakeholders after the planning stage. Several HCP identified staff turnover as an implementation barrier, especially at community hospitals. Champions had to train or engage new staff, and new champions had to be identified if established champions left the hospital.

Hospitals developed different methods for engaging key staff and eliminating implementation obstacles. HCP at some sites emphasized the importance of engaging surgeons as champions, while at other sites, HCP reported that empowering nurses was key to success. At academic hospitals, participants noted that implementing the bundle for inpatients required targeted and ongoing training for nurses and aides, close collaboration between inpatient staff and outpatient clinics, and specific strategies to inform colleagues about bundle steps and to document adherence. Several participants at one hospital reported missed opportunities because anesthesiology or surgical residents rotated off the service.

Few HCP reported activities related to bundle implementation that involved systematic reflection or evaluation. However, some reported that such activities could improve implementation. Given the lack of feedback or sustained engagement, 2 HCP expressed ambivalence about the value of continuing the intervention.

## Discussion

Our study identified key barriers and facilitators to bundle implementation for different surgical populations. Our findings have some important implications for the implementation of infection prevention bundles. Although sites agreed to implement a similar SSI prevention bundle, each hospital—and even different surgical areas within a hospital—implemented the protocol differently. Clinics and hospitals needed to adapt the bundle to their specific context, including workflow, patient population, and organizational culture.

Evidence-based recommendations can reduce the rate of preventable healthcare-associated infections including SSIs.^22,23^ Care bundles have reduced SSIs after some surgical procedures^8,9^ but not others.^13,24^ However, even evidence-based interventions like the STOP SSI bundle may not be implemented well, and HCP may not welcome the practice change. Although clinicians in our study valued both evidence and evidence-based practice, current infection rates shaped the willingness of some HCP to adopt or sustain the bundle. In other studies, hospitals have sometimes had difficulty adopting, sustaining, or complying with bundles.^25^ Hospitals have also had difficulty separating the efficacy of bundles or their constituent elements from each other, or from other infection control practices,^8,9,13,14,26^ potentially reducing their willingness to adopt bundles. Our findings agree with other reports about bundles, suggesting that implementation of bundles may face some common barriers across surgery types^23^ and that bundles require careful planning and ongoing strategies to sustain them. However, each setting may need to find different solutions to these barriers.

Our findings on the need to tailor and adapt interventions to specific settings resonate with implementation science insights in other contexts.^27,28^ The implementation of evidence-based bundles often requires refinements on a local level and may need to be adjusted specifically for the type of hospital and surgical unit and for inpatient and outpatient settings. Both community and academic hospitals participating in our study often adapted bundle elements and implementation strategies to fit their clinical contexts, and 2 academic hospitals pilot tested the bundle in specific areas to identify processes and helpful adaptations. Similarly, different surgical clinics faced specific hurdles (eg, integration of residents and fellows) that required bundle adaptation or specific facilitators. Even within specialties, providers sometimes described population-specific concerns (eg, different infection concerns for adult or pediatric patients having spine operations). Implementation tools can help identify helpful strategies for site-specific barriers.^19–21^

Similar to others studying implementation of evidence-based practices,^14,29–31^ we found that local champions facilitated successful adoption, planning, adaptation, and monitoring. However, in our study, the HCP who championed the bundle varied by setting. Additionally, interprofessional collaboration facilitated successful and smooth implementation of the bundle. Yanke et al^32^ found that implementation of a *C. difficile* infection control bundle was facilitated when HCP collaborated to identify barriers and facilitators. Hospitals interested in implementing an infection prevention bundle could identify all departments that should be directly or peripherally involved in the intervention and should facilitate early collaboration, engagement, and planning among representatives from each department. Interprofessional collaboration and champion engagement might circumvent the need for top-down approaches used by some HCP to push implementation.

Schweizer et al^15^ suggested previously that hardwiring steps into protocols or order sets facilitated implementation for the STOP SSI bundle. Hospitals committed to implementation should consider hardwiring elements as much as possible into departmental and hospital procedures (eg, protocols, order sets) to minimize unneeded variability among processes and units within the hospital, while allowing adaptation when needed. Hospitals that continually review the intervention and its constituent processes and work to simplify and adapt the intervention to local settings could improve their ability to sustain the intervention over time. Nevertheless, as shown in the implementation of an SSI-reduction bundle in colorectal surgery^33^ and our previous STOP SSI study,^15^ our study also suggests that bundle adherence is easier to ensure with outpatient elective surgeries. Implementing the STOP SSI bundle for patients undergoing urgent or emergent operations may require targeted adaptation and sustained interprofessional collaboration.

This study had several limitations. Some key implementers did not agree to be interviewed, and high turnover at community hospitals meant that some key implementers had moved to other hospitals. Thus, the perspectives of our key informants may have differed from the staff we could not interview. Additionally, not all hospitals or units implemented an identical bundle, limiting comparisons of all steps across all settings. Given our small sample size and the diversity of roles and settings represented, we did not feel it appropriate to report relative overall frequencies for findings. However, interviewing HCP in diverse roles allowed the integration of expert perspectives on bundle implementation at both community and academic hospitals.

In conclusion, while the STOP SSI bundle seems like a simple intervention, it was complicated to embed in various practice settings. In this qualitative study, hospitals and surgical services varied in their approaches to implementation and their strategies for overcoming obstacles. A single set of clearly defined implementation strategies for an intervention that intersects multiple departments and services in a hospital, as well as outpatient settings, may not be effective across all settings. Repeated evaluation and feedback might better inform appropriate adaptations for specific settings and populations and improve bundle implementation, fidelity, acceptability, and sustainment.
